# A randomized phase II study of paclitaxel alone versus paclitaxel plus sorafenib in second- and third-line treatment of patients with HER2-negative metastatic breast cancer (PASO)

**DOI:** 10.1186/s12885-017-3492-1

**Published:** 2017-07-25

**Authors:** Thomas Decker, Friedrich Overkamp, Siegfried Rösel, Arnd Nusch, Thomas Göhler, Martin Indorf, Jörg Sahlmann, Tanja Trarbach

**Affiliations:** 1 Onkologie Ravensburg, Ravensburg, Germany; 2 Oncologianova GmbH, Recklinghausen, Germany; 3 Onkodoc, Gütersloh, Germany; 4 Practice for Haematology and internal Oncology, Velbert, Germany; 5 Oncologic practice, Dresden, Germany; 6 iOMEDICO, Freiburg, Germany; 7 Center for Tumor Biology and Integrative Medicine Clinics Wilhelmshaven, Wilhelmshaven, Germany

**Keywords:** Metastatic breast cancer, Paclitaxel, Sorafenib, Antineoplastic agents/chemotherapy

## Abstract

**Background:**

We conducted an open-label, randomized, two-arm multi-center study to assess the efficacy and safety of paclitaxel versus paclitaxel + sorafenib in patients with locally advanced or metastatic HER2-negative breast cancer.

**Methods:**

Patients were randomly assigned to receive either paclitaxel monotherapy (80 mg/m^2^) weekly (3 weeks on, 1 week off) plus sorafenib 400 mg orally, twice a day taken continuously throughout 28 day cycles. Sorafenib dose was gradually escalated from a starting dose of 200 mg twice a day. The primary endpoint was progression free survival (PFS).

**Results:**

A pre-planned efficacy interim analysis was performed on the data of 60 patients, 30 patients in each treatment arm. Median PFS was estimated at 6.6 months (95% CI: 5.1 to 9.0) in patients randomized to single-agent paclitaxel (Arm A) and 5.6 months (95% CI: 3.8 to 6.5) in patients randomized to paclitaxel-sorafenib combination (Arm B) therapy. Contrary to the hypothesis, the treatment effect was statistically significant in favor of paclitaxel monotherapy (hazard ratio 1.80, 95% CI: 1.02 to 3.20; log-rank test *P* = 0.0409). It was decided to stop the trial early for futility. Median OS was also in favor of Arm A (20.7 months (95% CI: 16.4 to 26.7) versus 12.1 months (95% CI: 5.8 to 20.4) in Arm B. Clinical control was achieved in 28 patients (93.3%) in Arm A and in 21 patients 70.0% in Arm B. Overall response rate was met in 43.3% of patients in Arm A and in 40.0% in Arm B. Toxicities were increased in Arm B with higher rates of diarrhea, nausea, neutropenia, hand-foot skin reaction (HFSR) and anorexia, Grad 3 and 4 toxicities were rare.

**Conclusions:**

In this pre-planned interim analysis, paclitaxel-sorafenib combination therapy was not found to be superior to paclitaxel monotherapy with regard to the primary end point, progression-free survival. The trial was therefore discontinued early. There was no indication of more favorable outcomes for combination therapy in secondary efficacy end points. As expected, the safety and toxicity profile of the combination therapy was less favorable compared to monotherapy. Overall, this trial did not demonstrate that adding sorafenib to second- or third-line paclitaxel provides any clinical benefit to patients with HER2-negative advanced or metastatic breast cancer. Cautious dosing using a sorafenib ramp up schedule might have contributed to negative results.

**Trial registration:**

The study was registered at EudraCT (No 2009–018025-73) and retrospectively registered at Clinical trials.gov on March 17, 2011 (NCT01320111).

## Background

Breast cancer accounts for the greatest number of all new cases of cancer in women both in America and in Europe. Whereas the number of new cases of breast cancer has been increasing since 1980, mortality has been slightly decreasing in part because of improvements in primary treatment modalities [1, 2].

Nevertheless, once metastatic disease is established, the therapeutic strategy is seldom curative with median survival ranging from 2 to 3 years [3]. Recently, substantial progress has been made in HER2-positive disease [4, 5] and in overcoming endocrine resistance [6], but when it comes to the application of chemotherapy in HER2-negative disease, sequential use of single agents is still the therapy of choice because of increased toxicities in combination regimens [7].

The choice of the first line agent depends on previous adjuvant treatment, the need to induce a remission and patient preference regarding possible side effects. Taxans or anthracyclines are considered to be a standard of care, but a variety of other agents are frequently given because of toxicity or pretreatment issues. Disease control can be achieved in the majority of patients albeit for a limited amount of time [8].

Attempts to improve these results by adding agents targeting angiogenesis have been modestly successful. Bevacizumab, an antiangiogenic antibody, improves tumor response and progression free survival (PFS) when added to a variety of chemotherapeutic agents in the first-line [9] or second-line setting [10]. Similar results have been recently published with ramucirumab, another monoclonal antibody targeting the VEGF pathway [11].

Although chemotherapy is usually administered in the second-line setting, response rates are low with overall survival being less than 18 months [10, 12].

Sorafenib (Nexavar®) is a multikinase inhibitor with antiangiogenic and antiproliferative properties and is widely used in the treatment of advanced hepatocellular and renal cell carcinomas. Although clinical significant improvements were reported, toxicities were substantial [13, 14].

Preclinical data in breast cancer models have shown additive/synergistic effects by adding sorafenib to cytotoxic drugs [15, 16]. Early studies demonstrated limited efficacy but reasonable tolerability of sorafenib when used as a single agent in metastatic breast cancer [17]. However, promising results were published when sorafenib was combined with capecitabine in first-line or second-line treatment in a randomized phase IIb trial. Whereas overall survival and objective response rates were not improved, a significant and potentially clinical relevant difference in PFS was described [18]. Because of overlapping toxicities (hand-foot skin reaction (HFSR), diarrhea), the combination of capecitabine and sorafenib resulted in unacceptable toxicities. Combined treatment with sorafenib and paclitaxel was also tested in the first line setting in a phase IIb trial. Although the primary endpoint (prolongation of PFS) was not met, disease control was improved. Toxicities were substantial but manageable with dose reductions [19].

Therefore, we initiated this trial of sorafenib in combination with paclitaxel and started with a sorafenib dose of 200 mg bid which was gradually escalated to 400 mg bid within the first 3 cycles to avoid early toxicities. The safety and efficacy results of a pre-planned interim analysis of the PASO trial in patients receiving second- or third-line treatment are reported here.

## Methods

Written informed consent was given by all patients before enrolment. All patients had to meet the following inclusion criteria: Patients were female and age 18 or older, with HER-2-negative locally recurrent (inoperable) or metastatic breast cancer with an indication for second- or third-line chemotherapy. An Eastern Cooperative Oncology Group (ECOG) score of 0 or 1 was required. Prior endocrine or radiation therapy was allowed as well as treatment with taxanes in the neoadjuvant or adjuvant setting. Pretreatment with bevacizumab was also allowed. Anticancer chemotherapy, hormontherapy, immunotherapy or radiotherapy had to be terminated at least 3 weeks prior to study entry. Patients were ineligible if they had known brain metastasis, inadequate bone marrow, liver or kidney function, previous or concurrent cancer, or had a significant risk for major cardiovascular or cerebrovascular events. HER2 status was determined using ICH or FISH/CISH test. Response was evaluated based on RECIST 1.1 by the investigators. Patients were followed up for toxicity, progression free survival and death until 12 months after the last patient ended study treatment.

All procedures performed in this study were in accordance with the ethical standards of the institutional and/or national research committee and with the 1964 Helsinki declaration and its later amendments or comparable ethical standards. The study was sponsored by GMIHO.

### Study design

PASO was an open-label, randomized, two arm multi-center study to assess the efficacy and safety of paclitaxel (Arm A) versus paclitaxel + sorafenib (Arm B) in patients with locally advanced or metastatic HER2-negative breast cancer**.** The study was conducted in 21 centers in Germany. Patients were randomly assigned to the respective treatment arms. Prior to randomization, patients were stratified by second- and third-line treatment as well as by possible pre-treatment with bevacizumab. Randomization was performed in a 1:1 ratio using sequential numbers, which were randomly allocated to one of the treatment arms. Paclitaxel was given at 80 mg/m^2^ as one-hour i.v. infusion on day 1, 8, 15 and sorafenib 400 mg orally twice daily (bid) taken continuously throughout 28-day cycles. Treatment was administered until tumor progression or until unacceptable toxicity. Sorafenib was to be started as 200 mg in the morning and 200 mg in the evening (cycle 1), increased to 200 mg in the morning and 400 mg in the evening (cycle 2) and further increased to the maintenance dose of 400 mg in the morning and 400 mg in the evening (from cycle 3). Specific dose reduction schedules were recommended for hematological/non-hematological toxicities and HFSR.

The primary endpoint was progression free survival (PFS). Secondary endpoints included time to progression (TTP), time to next treatment (TTT), clinical control (complete response (CR) + partial response (PR) + stable disease (SD)) and overall response rate (CR + PR).

Adverse events (AEs) were graded according to the National Cancer Institute Common Terminology Criteria for Adverse Events (NCI-CTCAE), version 4.02.

### Statistical analysis

The sample size for this study was determined based on the assumption that the median PFS for patients receiving paclitaxel in first-line treatment is 5.6 months [19]. As patients in second- and third-line treatment were included in the study, a PFS of 4 months was assumed for the control arm (single-agent paclitaxel). Improvement by 50% to a median PFS of 6 months in the test arm (paclitaxel-sorafenib combination) was considered to be clinically significant. A total of 110 events were required to detect a difference in median PFS of 4 vs. 6 months (one-sided test, α = 0.1, 80% power). Assuming a recruitment period of 12 months and a follow-up period of a further 12 months, a total sample size of 122 patients was required. To account for a drop-out rate of about 15% a total of 140 patients were planned to be randomized.

Analysis of efficacy was conducted based on the intent-to-treat (ITT) principle. PFS was estimated using time-to-event analysis by Kaplan and Meier. Kaplan-Meier estimators were presented as survival curves by treatment arm with 95% confidence intervals (CIs); median PFS times were computed with 95% CIs for both treatment arms.

The two treatment arms were compared with respect to PFS using a one-sided log-rank test at a level of significance of α = 0.1 (primary end point). All other statistical analyses of efficacy parameters were descriptive in nature, i.e. the results of comparisons were to be interpreted in an exploratory manner for the generation of hypotheses. Unless specified otherwise, those results were to be reported without giving the significance level.

## Results

Between August 2010 and November 2012, 61 patients were randomly assigned to treatment. Data cutoff for analysis of the primary endpoint PFS as well as secondary endpoints including OS was July 2014. The full analysis set (ITT set), which was used for efficacy analysis includes 60 patients, with 30 patients in each treatment arm. The safety set includes 57 patients who received any study medication, 29 in Arm A and 28 in Arm B.

### Baseline characteristics

Baseline characteristics are presented in Table 1. Mean (median) age was 59.4 (58) years in Arm A and 59.0 (62) years in Arm B. At least 2 organs were affected by metastases in 16 patients (53%) and 21 patients (70%), respectively. Based on a decision rule combining all assessable data of several test methods, only 1 patient (Arm A) was definitely HER2-positive. Most patients were postmenopausal (Arm A: 70%, Arm B: 83%), and tumors often expressed hormone receptors with 17.7% and 20% of triple negative tumors in Arm A and B, respectively. The percentage of patients with at least two affected organs was higher in the experimental arm (70% versus 53.3%).

**Table 1:** Patient characteristics at enrolment in Arm A vs. Arm B

	Arm A Paclitaxel *N* = 30	Arm B Paclitaxel + Sorafenib *N* = 30
Age, mean (SD), years	59.4 (10.4)	59.0 (9.6)
ECOG status, n (%)		
0	23 (76%)	19 (61%)
I	7 (24%)	11 (39%)
Number of metastatic sites		
< 2	14 (46.7)	9 (30)
> = 2	16 (53.3)	21 (70)
Hormone receptor status, n (%)		
ER+ or PR+	25 (83.3)	24 (80.0)
ER- and PR-	5 (16.7)	6 (20.0)
Pre-treatment with bevacizumab		
No	7 (23.3)	8 (26.7)
Yes	23 (76.7)	22 (73.3)
Line of therapy, n (%)		
Second-line	20 (66.6)	21 (70.0)
Third-line	10 (33.3)	9 (30.0)
Taxan pretreatment n (%)		
No	23 (76.7)	20 (66.7)
Yes	7 (23.3)	10 (33.3)

In more than 2/3 of patients, study medication was administered as second-line therapy (Arm A: 67%, Arm B: 70%); the other patients received third-line therapy. Previous therapy with taxanes in the (neo-)adjuvant setting included docetaxel and paclitaxel (in total: Arm A: 23.3%, Arm B: 33.3%). Most patients in both arms were pretreated with capecitabine or vinorelbine in the metastatic setting. About three quarters of patients had received previous therapy with bevacizumab (Arm A: 77%, Arm B: 73%). More patients had an ECOG performance status of 0 in Arm A (76%) versus 61% in Arm B.

### Efficacy

**Fig. 1:**
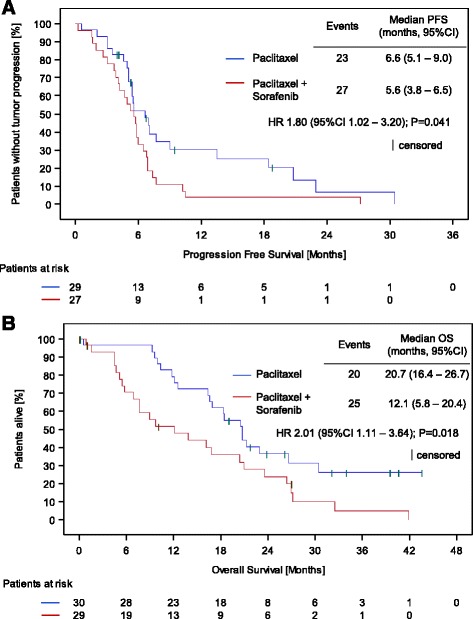
Kaplan-Meier estimates of (**a**) the primary endpoint of progression free survival and (**b**) the secondary endpoint of overall survival (OS)

A pre-planned interim analysis was performed on the data of 60 patients, 30 patients in each treatment arm. Disease progression or death was documented for 23 patients (76.7%) in Arm A and for 27 patients (90.0%) in Arm B. Median PFS was 6.6 months (95% CI: 5.1 to 9.0) in Arm A and 5.6 months (95% CI: 3.8 to 6.5) in Arm B (Fig. 1a). The confirmatory test for the effect of treatment on PFS was statistically significant (hazard ratio 1.80, 95% CI: 1.02 to 3.20; log-rank test *P* = 0.041). Contrary to the hypothesis, the treatment effect was in favor of paclitaxel monotherapy. There was no effect of the covariates on PFS, neither for previous therapy with bevacizumab nor for therapy line. It was decided to stop the trial early for futility of demonstrating the superiority of paclitaxel-sorafenib combination therapy.

Death was documented for 20 patients (66.7%) in Arm A and for 25 patients (83.3%) in Arm B. Median OS was 20.7 months (95% CI: 16.4 to 26.7) in patients randomized to single-agent paclitaxel and 12.1 months (95% CI: 5.8 to 20.4) in patients randomized to paclitaxel-sorafenib combination therapy (Fig. 1b). The exploratory test for a treatment effect on OS was significant (hazard ratio 2.01, 95% CI: 1.11 to 3.64; *P* = 0.018), indicating a treatment effect on OS in favor of paclitaxel monotherapy. However, this result may be partly due to 3 outliers whose life expectancy was markedly shorter than required by a major inclusion criterion (1 patient in Arm A: 17 days; 2 patients in Arm B: 27 and 48 days).

Median TTP was 6.6 months (95% CI: 5.1 to 9.0) in Arm A and 5.3 months (95% CI: 3.8 to 6.5) in Arm B. The exploratory test for a treatment effect on TTP was significant (hazard ratio 1.98, 95% CI: 1.06 to 3.71; *P* = 0.030), indicating a treatment effect on TTP in favor of paclitaxel monotherapy.

Next treatment was documented for 27 patients (90.0%) in Arm A and 17 patients (56.7%) in Arm B. Median TTT was estimated at 7.4 months in patients randomized to single-agent paclitaxel and 7.0 months in patients randomized to paclitaxel-sorafenib combination therapy. The exploratory test for a treatment effect on TTT was not significant (hazard ratio 1.38, 95% CI: 0.72 to 2.63; *P* = 0.334).

Clinical control was achieved in 28 patients (93.3%) in Arm A and in 21 patients (70.0%) in Arm B, suggesting a difference in favor of single-agent paclitaxel (*P* < 0.05).

There was no statistical difference between treatment arms for ORR 43.3% in Arm A and 40.0% in Arm B. The investigator assessed efficacy outcomes are shown in Table 2.

**Table 2:** Investigator assessed efficacy endpoints in arm A and arm B

Endpoint	Arm A	Arm B		
	Median (95% CI)	Median (95% CI)	HR	*P* value
Progression-free survival (months)	6.6 (5.1–9.0)	5.6 (3.8–6.5)	1.80	0.041
Overall survival (months)	20.7 (16.4–26.7)	12.1 (5.8–20.4)	2.01	0.018
Time to Progression (months)	6.6 (5.1–9.0)	5.3 (3.8–6.5)	1.98	0.030
Time to next treatment (months)	7.4 (6.1–10.4)	7.0 (5.8–10.3)	1.38	0.334
	n(%);(95% CI)	n(%);(95% CI)		*P* value
Clinical control rate	28 (93.3) (77.6–99.2)	21 (70.0) (52.0–83.5)		0.020
Overall response rate	13 (43.3) (27.4–60.8)	12 (40) (24.6–57.7)		0.793

### Safety results

29 patients in Arm A and 28 patients in Arm B constitute the safety population. The median number of cycles administered (completed or started) was 6 cycles per patient in Arm A and 4 cycles per patient in Arm B.

Apart from 1 patient in Arm A, all patients experienced at least 1 AE. The most common AEs of any toxicity grade in Arm A were peripheral neuropathy (59%), fatigue (45%), diarrhea (41%) and alopecia (41%). In Arm B, the most common adverse events were diarrhea (57%), fatigue (50%), oral mucositis (43%), anorexia (43%) and peripheral neuropathy (39%).Table 3 summarizes AE rates occurring in >20% of patients for all toxicity grades.

**Table 3:** Adverse events reported in at least 20% of patients of any (safety population)

Event (n(%)) System organ class Preferred term	Arm A (*n* = 29)		Arm B (*n* = 28)	
	All Grades	Grade 3/4	All Grades	Grade 3/4
Blood and lymphatic system disorders	7 (24.1%)	1 (3.4%)	7 (25.0%)	3 (10.7%)
Anemia	6 (20.7%)	1 (3.4%)	6 (21.4%)	2 (7.1%)
Gastrointestinal disorders	22 (75.9%)	1 (3.4%)	26 (92.9%)	7 (25.0%)
Constipation	5 (17.2%)	-	6 (21.4%)	-
Diarrhea	12 (41.4%)	-	16 (57.1%)	3 (10.7%)
Mucositis oral	9 (31.0%)	-	12 (42.9%)	-
Nausea	6 (20.7%)	-	9 (32.1%)	2 (7.1%)
General disorders and administration site conditions	20 (69.0%)	1 (3.4%)	22 (78.6%)	3 (10.7%)
Edema limbs	8 (27.6%)	-	1 (3.6%)	-
Fatigue	13 (44.8%)	-	14 (50.0%)	-
Pain	8 (27.6%)	-	9 (32.1%)	1 (3.6%)
Investigations	7 (24.1%)	1 (3.4%)	14 (50.0%)	9 (32.1%)
White blood cell decreased	3 (10.3%)	1 (3.4%)	12 (42.9%)	7 (25.0%)
Metabolism and nutrition disorders	3 (10.3%)	1 (3.4%)	14 (50.0%)	1 (3.6%)
Anorexia	1 (3.4%)	-	12 (42.9%)	1 (3.6%)
Nervous system disorders	21 (72.4%)	3 (10.3%)	18 (64.3%)	6 (21.4%)
Dizziness	6 (20.7%)	-	1 (3.6%)	-
Dysgeusia	1 (3.4%)	-	7 (25.0%)	-
Peripheral sensory neuropathy	17 (58.6%)	2 (6.9%)	11 (39.3%)	4 (14.3%)
Respiratory, thoracic and mediastinal disorders	14 (48.3%)	1 (3.4%)	17 (60.7%)	2 (7.1%)
Epistaxis	6 (20.7%)	-	7 (25.0%)	-
Skin and subcutaneous tissue disorders	15 (51.7%)	-	20 (71.4%)	3 (10.7%)
Alopecia	12 (41.4%)	-	6 (21.4%)	-

In Arm A, 8 patients (28%) experienced grade 3 events. Except for peripheral sensory neuropathy in 2 patients (7%), all grade 3 symptoms occurred in 1 patient (3.4%). No grade 4 events were reported in Arm A. In Arm B, 24 patients (86%) experienced grade 3 events. Of these, the incidence was highest for leukopenia (25%), peripheral sensory neuropathy (14%) and diarrhea (11%). Grade 4 events in Arm B were reported for 3 patients (11%); each of the following symptoms was reported in 1 patient (4%): nausea, white blood cell decreased (leukopenia) and thrombocytopenia. Of note, the incidence of hand-foot syndrome was rather low in Arm B with 14% events of any grade and 7% grade 3 events.

In Arm A 2% (*n* = 22) of planned paclitaxel applications (*n* = 550) were not administered (postponed: 5.6%, *n* = 31; dose modified: 4.0%, *n* = 22). In Arm B 13.8% (*n* = 55) of planned paclitaxel applications (*n* = 399) were not administered (postponed: 8.8%, *n* = 35; dose modified: 6.0%, *n* = 24). Treatment with paclitaxel was permanently discontinued due to adverse events in 11 patients (38%) in Arm A and 14 patients (50%) in Arm B.

According to the ramp up schedule of the study protocol, the mean daily dose of sorafenib was increased during the first 3 cycles (mean daily dose of 407 mg during cycle 1, 545 mg during cycle and 629 mg during cycle 3). However, dose reductions were frequently needed in Arm B with at least one dose modification in 19 patients (67.9%).

## Discussion

There is an unmet medical need for new therapeutic strategies that are effective in reducing the risk of disease progression and early death for patients with advanced or recurrent metastatic breast cancer. Although many lines of chemotherapy are commonly administered, the actual benefit of treatment for patients receiving second- and further-line therapy are hard to quantify [20] and only limited progress has been reported in this setting [21].

Attempts to improve results by combining chemotherapy with antiangiogenic drugs targeting the VEGF pathway have resulted in conflicting results. In general, modest gains in progression free survival and response rates did not translate into improved overall survival [9].

Sorafenib is a potent multikinase inhibitor with antiangiogenic and antiproliferative properties and therefore has a broader spectrum of activity than bevacizumab. It does not only target the VEGF-2 receptor, but also the platelet-derived growth factor receptor-ß, c-Kit, Flt and Raf kinase [22].

The objective of PASO, a randomized controlled phase II study, was to demonstrate the superiority of paclitaxel-sorafenib combination therapy over paclitaxel monotherapy in second- or third-line treatment of patients with HER2-negative locally advanced or metastatic breast cancer. However, the trial was terminated early after a pre-planned interim analysis has shown that the treatment effect was statistically in favor of paclitaxel monotherapy.

In the PASO trial, paclitaxel was chosen because of non-overlapping toxicities with sorafenib and its established clinical efficacy in metastatic breast cancer. Although current guidelines recommend taxan-based chemotherapy early in the course of the disease [8], taxanes are often used in clinical practice in later treatment lines to avoid alopecia and long standing neuropathy (N. Marschner, manuscript in preparation). Combination of chemotherapy and sorafenib has been reported to result in additional toxicities and frequent dose interruptions and reductions [18, 23]. To avoid excessive toxicities of the combination therapy, sorafenib dose was gradually increased according to a prespecified ramp up schedule. Using this schedule, sorafenib dose could be increased gradually from 407 mg during cycle 1 to 630 mg in cycle 3, with frequent doses modifications due to toxicity. However, toxicities were rather modestly increased in patients receiving combination therapy with increases in leukopenia, mucositis and diarrhea. Especially the incidence of grade 3 HFSR was rather low compared to the incidence of HFSR with paclitaxel and sorafenib started at full dose [19]. Importantly, early occurrence of HFSR has been described to be a predictive factor for tumor control in hepatocellular carcinoma patients [24, 25].

In order to manage or prevent toxicities, the investigators of the PASO trial did not fully utilize the recommended dose of 800 mg (400 mg twice daily). As a result of such cautious dosing, a number of patients received sorafenib in a potentially subtherapeutic range, with a possible impact on clinical efficacy outcomes. A similar dose escalation strategy was reported in a neoadjuvant phase II trial. Cumulative sorafenib doses between 37% and 65% of the recommended dose of 800 mg daily were given depending on the treatment schedule [26].

In an attempt to alleviate the toxicities of the capecitabine and sorafenib combination, the phase III RESILIENCE trial also started with a lower sorafenib dose, which could be escalated to 400 mg BID if tolerated. In addition, detailed guidelines for prophylactic and symptomatic treatment were provided [27]. In contrast to promising data from the phase II SOLTI trial [18] the addition of sorafenib to capecitabine did not meet the primary endpoint of prolonging progression free survival. There even was a trend for lower overall survival. The reduced starting dose of Sorafenib might have contributed to these inferior outcomes as in our trial [28]. Hypothetically, the growth enhancing effect of sorafenib at lower doses might have contributed to these results [29].

Indeed, treatment with sorafenib should be initiated with the approved dose of 400 mg bid in hepatocellular cancer as evidence is lacking from real world non interventional studies that lower starting doses result in equivalent outcomes [30]. In addition, there was a trend of reduced OS survival in patients with differentiated thyroid cancer treated with a lower sorafenib dose (30 versus 56 months, *p* = 0.08) [31].

The reduced starting dose could therefore explain the inferior outcome in our study as compared to the study by Gradishar et al. [19]. Addition of sorafenib to paclitaxel improved disease control and overall response in this study although PFS or OS were not significantly prolonged. Importantly, sorafenib was started full dose at 400 mg bid. In addition, while treatment delays and dose reductions are only slightly more frequent in Arm B, paclitaxel was not administered much more often in Arm B. These differences imply that in addition to a reduced sorafenib dosing, also paclitaxel application was not conducted according to the preplanned schedule. This might also have contributed to a superior result of Arm A.

Although the two treatment groups were generally balanced with respect to their clinical characteristics as assessed at screening, some negative prognostic factors were not evenly attributed. First, lung, thorax and brain were more frequently affected by metastases in Arm B than in Arm A. Second, in patients receiving combination therapy, there appeared to be more organs affected: At least 2 organs in 21 patients (70%) compared with 16 patients (53%) who received standard therapy. Third, patients who also received sorafenib tended to be more impaired in activities of daily living. At screening, all patients treated had a Karnofski performance status ≥70%, i.e. were ECOG 0 or 1. However, the proportion of patients who were restricted in physically strenuous activity (ECOG 1) was higher in Arm B: 11 patients (39%) versus 7 patients (24%) in Arm A. In addition, previous treatment with paclitaxel or docetaxel as part of adjuvant or neoadjuvant therapy was more frequent in patients in the experimental arm (33,3% vs 23,3%). In view of the small sample, the numerical disparity was not explored statistically, but an influence on outcome cannot be excluded. A large number of patients were pretreated with bevacizumab in our trial (more than 70% in both treatment arms). This raises the concern of a possible cross resistance between bevacizumab and sorafenib, which both target the VEGF receptor pathway, However, clinical data in renal cancer demonstrates that treatment with the multi-kinase inhibitor sunitinib is efficacious in patients previously treated with bevacizumab [32]. In addition, continued targeting of the VEGF receptor pathway beyond progression was associated with clinical benefit in a variety of cancer types including breast cancer [33, 34].

Another limitation of our study is related to design. For reasons of practicality, it was conducted open label without placebo added to paclitaxel in Arm A. Moreover, tumor response was evaluated by the investigators and not additionally by blinded independent centralized review. However, it is regarded as very unlikely that such design features would have impacted the overall conclusions.

## Conclusions

In summary, the PASO trial adds data to the growing evidence, that addition of sorafenib to chemotherapy in unselected patients with metastatic breast cancer should not be further explored as a therapeutic strategy in this group of patients. The ramp up design resulted in a favorable toxicity but the inferior outcome in the combination arm raises the question of “starting right”. In the absence of prospective randomized data, reduced starting doses of targeted agents should not be routinely administered.

## Abbreviations

AEs: Adverse events
CI: Confidence interval
CR: Complete response
ECOG: Eastern cooperative oncology group
HER2: Human epidermal growth factor receptor 2
HFSR: Hand-foot skin reaction
ITT: Intent-to treat
NCI-CTCAE: National Cancer Institute Common Terminology Criteria for Adverse Events
ORR: Overall response rate
OS: Overall survival
PFS: Progression free survival
PR: Partial response
TTP: Time to progression
TTT: Time to next treatment
VEGF: Vascular endothelial growth factor
WBC: White blood cell
